# Exceptional performance with minimal data using a generative adversarial network for alzheimer's disease classification

**DOI:** 10.1038/s41598-024-66874-5

**Published:** 2024-07-24

**Authors:** Pui Ching Wong, Shahrum Shah Abdullah, Mohd Ibrahim Shapiai

**Affiliations:** 1 Biologically Inspired System and Technology Laboratory, Department of Electronic Systems Engineering, Malaysia-Japan International Institute of Technology, Universiti Teknologi Malaysia, Kuala Lumpur, Malaysia; 2 Centre for Artificial Intelligence and Robotics Laboratory, Department of Electronic Systems Engineering, Malaysia-Japan International Institute of Technology, Universiti Teknologi Malaysia, Kuala Lumpur, Malaysia

**Keywords:** Alzheimer's disease, Electrical and electronic engineering

## Abstract

The classification of Alzheimer's disease (AD) using deep learning models is hindered by the limited availability of data. Medical image datasets are scarce due to stringent regulations on patient privacy, preventing their widespread use in research. Moreover, although open-access databases such as the Open Access Series of Imaging Studies (OASIS) are available publicly for providing medical image data for research, they often suffer from imbalanced classes. Thus, to address the issue of insufficient data, this study proposes the integration of a generative adversarial network (GAN) that can achieve comparable accuracy with a reduced data requirement. GANs are unsupervised deep learning networks commonly used for data augmentation that generate high-quality synthetic data to overcome data scarcity. Experimental data from the OASIS database are used in this research to train the GAN model in generating synthetic MRI data before being included in a pretrained convolutional neural network (CNN) model for multistage AD classification. As a result, this study has demonstrated that a multistage AD classification accuracy above 80% can be achieved even with a reduced dataset. The exceptional performance of GANs positions them as a solution for overcoming the challenge of insufficient data in AD classification.

## Introduction

Alzheimer's disease (AD) is a chronic neurological disease associated with the deterioration of the brain's cognitive abilities. It usually affects older adults whose common symptoms include memory loss, unresponsiveness, gradual impairment of language function, and gradual decline in daily living abilities. Research has shown that AD is the most common form of dementia, accounting for 60% to 80% of patients with dementia^1^. Moreover, it is also known as an irreversible disease due to its progressive nature and lack of cure ﻿at present^2^. Current treatments and medicines can help alleviate symptoms and slow disease progression only. Thus, early detection and diagnosis of AD are highly important for delaying the worsening of this incurable neurodegenerative disease.

Medical imaging techniques are commonly used to detect and diagnose AD. Numerous methods, such as magnetic resonance imaging (MRI), positron emission tomography (PET), and computed tomography (CT), are available. However, MRI is the most popular medical imaging technique used for diagnosing AD due to its superior suitability. According to related research, patients are more likely to undergo MRI scans than PET and CT scans due to concerns about safety and price issues^3^. MRI uses magnetic fields and radio waves to scan the inside of the brain, but unlike PET and CT, it does not emit radiation to the brain. In addition, an MRI scan is also not as expensive as a PET scan, thus leading to its use in AD diagnosis.

However, the abundance of images produced by MRI scans presents a significant challenge for clinicians and doctors in analyzing such extensive data. Recognizing the limitations of manual analysis, which is both time-consuming and prone to errors^4^, there is growing interest in computer-aided diagnosis methods^5^. For instance, research indicates that machine learning approaches have the potential to enhance the efficiency and accuracy of image analysis processes. Salunkhe et al.^6^ tested the effectiveness of 3 different machine learning techniques for diagnosing AD using MRI images. Support vector machine (SVM), random forest (RF), and decision tree (DT) models were used to classify the data. As a result, the best-performing classifiers identified were the ensemble model (90.2%), the DT model (88.5%), and the SVM model (87.2%). Thus, this study proves the effectiveness of machine learning methods.

Nevertheless, when dealing with more significant amounts of data, research shows that machine learning methods tend to encounter challenges such as decreased performance due to the complexity of MRI images^7^. Therefore, deep learning techniques have been gradually gaining attention as replacements for existing methods that require advanced engineering techniques and specialized expertise to handle large datasets. In a study carried out by Jain et al.^8^, the authors used a CNN model to classify MRI images in multiple stages of AD and achieved a high accuracy of 95.73%. In general, multistage classification of ADs is more challenging than binary classification tasks due to the difficulty in detecting prodromal stages that tend to have less obvious features. Thus, by using deep learning techniques such as those described in^8^, MRI has been shown to increase the performance of diagnosing AD, leading to the recent trend in this field of research.

While CNNs can achieve better performance in classification tasks, challenges arise, such as an insufficient amount of training data^9^. However, deep learning models require large amounts of data to learn and perform classification tasks^10^. On the other hand, medical image data, such as MRI images, are scarce due to patient privacy issues, which are heavily protected. Appropriate consent and applications are needed to access patients' medical records to obtain data for research purposes. Therefore, the problem of insufficient data has arisen when training deep learning models for AD classification.

Generative data augmentation techniques such as the generative adversarial network (GAN) seem to be useful for addressing this issue, given their great application in image generation tasks. It can generate realistic artificial samples of medical images^11^, overcoming the limitations of transformation-based techniques^12^. Thus, the current research trend is to apply a GAN, a type of unsupervised deep learning model, to solve the problem of insufficient data. Cabreza et al.^13^ utilized a deep convolutional GAN to solve the issue of insufficient labeled data for anomaly detection on MRI images to diagnose AD. As a result, an accuracy of 74.44% was achieved. In addition, focusing on small memory requirements, Jung et al.^14^ adopted another type of GAN known as the conditional GAN for generating synthetic MR images in different stages of AD. Thus, these existing studies infer that the GAN is useful for expanding small medical image datasets to solve the problem of insufficient medical data.

Based on the great achievements of GANs in medical image generation tasks, this research aims to propose a GAN-based method that can achieve the same classification accuracy with less data by generating additional synthetic MR images. Unlike existing studies, this research focuses on using fewer data points to achieve the same accuracy instead of expanding the dataset. Since the problem of insufficient MRI data has already been solved, this study aimed to adapt to this situation by using less data for training with the help of a GAN. In other words, this research seeks to maintain the exceptional performance of deep learning techniques in diagnosing AD while utilizing less MRI data for training the model. With this approach, the issue of insufficient data can be omitted if the deep learning model can achieve the same classification accuracy with less data.

## Methodology

### Datasets

This study used an open-access database called the Open Access Series of Imaging Studies (OASIS) to obtain MRI images for training deep learning models. OASIS is a commonly used public database in the scientific community for healthy aging and dementia-based research and studies^15^. Three (3) series of datasets have been released since its establishment in 2007, with the latest release of the OASIS-3 dataset. The OASIS-3 is a collection of MRI and PET images from 1098 participants and their related clinical data. All the data are obtained from ongoing projects at the Washington University Knight Alzheimer Disease Research Centre for 15 years. A total of 1098 participants comprised 605 cognitively healthy adults and 493 people with different stages of cognitive decline, while their ages ranged from 42 to 95 years. Nearly 2000 MR sessions with various structural and functional sequences are included in this database.

As this study aimed to use minimal data to achieve the same classification accuracy for AD diagnosis, only 300 MRI subjects were chosen from the OASIS-3 database for training the deep learning model. This research focuses on a multistage AD classification system in which the subjects will be classified into different stages of the disease: AD, MCI, and NC. Thus, a balanced dataset of MR images with 100 subjects per class will be obtained from the database to avoid bias in the results. Moreover, the acquisition parameters of the dataset for all 3 classes are also standardized to the usage of axial T1-weighted MR images scanned using a 3 Tesla scanner only. For such specifications in the OASIS-3 database, there are generally 256 slices of images produced per MR scan, illustrating the cross-sectional area of the brain.

**Figure 1:**
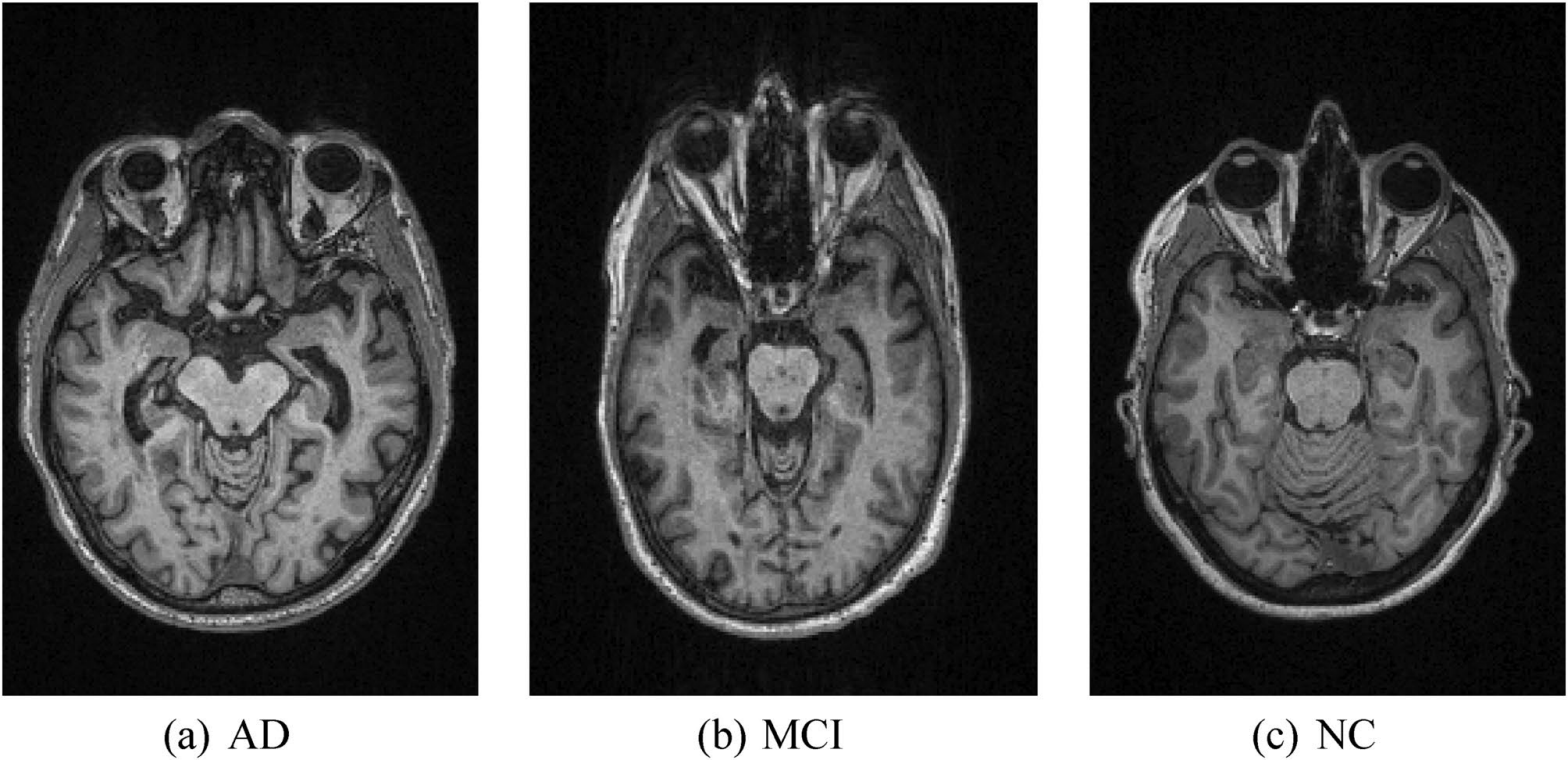
Examples of MR images from OASIS in different classes of AD.

Since this study utilizes only MRI data in axial view, the 256 MRI slices consist of images scanned from the top to bottom of the brain in an X–Y plane parallel to the ground. Hence, not all the slices contain clinically relevant information for this study. For instance, some slices might capture the areas outside the brain such as upper neck or skull, but such information is not important for AD classification. In identifying AD, the brain region known as the hippocampus is the most significant region needed for analysis. Thus, slices that do not contain information of the hippocampus region are also considered as irrelevant slices. In order to maintain the quality of images used for training the deep learning model, filtration of MRI slices is performed. Firstly, since the hippocampus region is located at the center part of the brain, the slices that captured the upper and lower part of the brain are irrelevant and are filtered out. Then, the remaining slices are manually filtered by observing the availability of the hippocampus region. Finally, after filtering out all the irrelevant slices experiencing hippocampal absence, each subject was left with only approximately 10 useful MRI slices. As a result, for each class that has 100 subjects, there will be around 1000 slices per class, resulting in a total of 3000 MRI slices used in training the GAN model. Figure 1 shows examples of MRI images obtained from the OASIS database for 3 different classes of AD.

Table 1 shows the demographic and clinical characteristics of the MRI subjects selected for this study. For the whole MRI dataset collected from the OASIS database, the sex and age distributions were standardized during data collection to avoid bias. Both male and female subjects were collected in the distribution of 44% versus 56% for all three classes, while the ages of the MRI subjects ranged from 65 years old to 74 years old, as shown in Table 1. Selection of subjects were based on clinical dementia rating (CDR), a widely used scale for assessing the severity of dementia symptoms. A CDR larger than 2 indicates the AD stage, a score between 0.5 to 1 indicates the MCI stage, while CDR equivalent to zero represents the NC stage. Lastly, all the MRI images obtained are in the same size of 256 pixels × 176 pixels.

**Table 1:** Demographic and clinical characteristics of the selected MRI subjects.

Classes	AD	MCI	NC
Number of subjects	100	100	100
Sex	Male: 44%	Male: 44%	Male: 44%
	Female: 56%	Female: 56%	Female: 56%
Age	69.5 ± 2.25	69.5 ± 2.25	69.5 ± 2.25
CDR	CDR > 2	0.5 < CDR < 1	CDR = 0
Number of MRI slices	1000	1000	1000
Size of MRI slices	256 pixels × 176 pixels	256 pixels × 176 pixels	256 pixels × 176 pixels

### Proposed method

**Figure 2:**
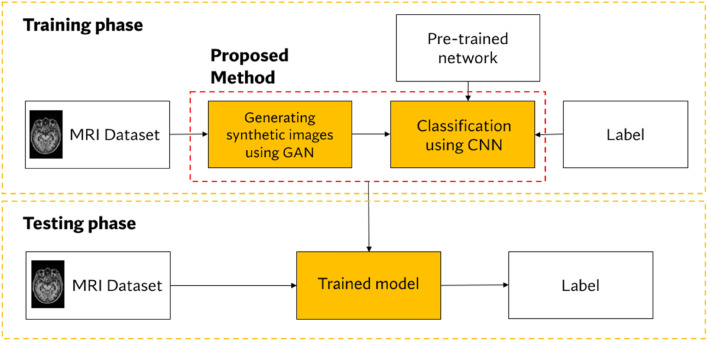
Overall framework of the proposed method.

The proposed methodology in this study consists of two steps: (1) generating synthetic images using a GAN and (2) performing multistage AD classification via deep learning networks. The overall framework is shown in Fig. 2, and the significant steps are discussed in the following subsections.

There are 2 phases in general: the training and testing phases involved in this proposed method. The proposed method will first be trained on the input MRI dataset in the training phase. After the training session, the trained model was tested on unseen MR images for generalizability. The ability of the model to accurately classify MRI images in their respective classes will be the benchmark of this study.

#### Generative adversarial network (GAN)

A GAN is a type of progressive deep learning network that is commonly used for generative tasks. Its architecture was first introduced by Goodfellow et al.^16^, and since then, it has been the most popular generative model due to its ability to generate realistic and high-quality images. It is mainly applied to solve data shortage and class imbalance problems^17^. In recent years, there has been a surge of studies utilizing GANs for generating medical images, such as brain MRI^18–20^. Using unsupervised learning, it learns and summarizes the probability distribution of input variables and then generates new samples based on its knowledge.

**Figure 3:**
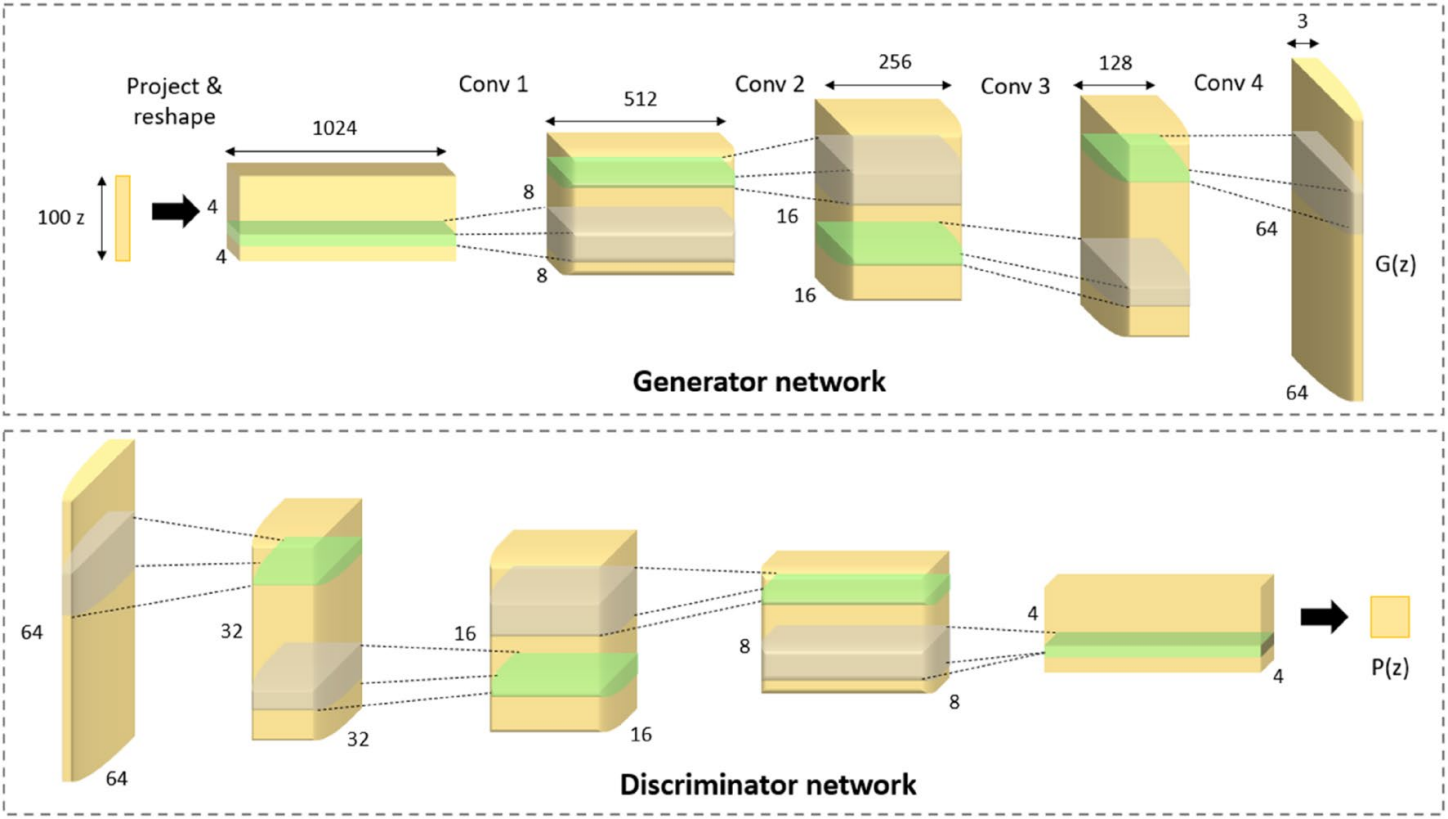
GAN model architecture^16^.

Figure 3 shows the model architecture of a GAN. It comprises two networks, which are the generator and discriminator. The generator generates new samples that look like the actual data as much as possible. On the other hand, the discriminator learns the data features and tries to differentiate the generated samples from the data. The mechanism of both networks is based on game theory, where one network tries to hide and the other seeks. The generator will continue updating its model and generate perfect samples that resemble real data by hiding any flaws. Moreover, the discriminator will be the "police" that seeks and detects fake-looking samples that do not look like real data. The training process of both models ends when equilibrium is achieved: the discriminator can no longer discriminate between the generated samples and the real data.

In this research, MR images from each class; AD, MCI, and NC obtained from the OASIS database are inputted into the GAN model to generate synthetic MR images for experimental purposes. The MRI data collected is divided into 3 separate datasets systematically according to their classes and the GAN model is trained on the 3 datasets sequentially. In the training loop, the GAN model iterates over the data loaders for each class and trains for a certain number of epochs. The generator generates sample MRI images for each class while the discriminator performs binary classification on the samples generated by classifying whether they are real or fake images. If the samples are classified as real images, the discriminator labels them as one (1), while fake images are labeled 0. Then, after looping through all the three datasets, the generated images are saved to a directory specific to each class with folders labelled as “AD”, “MCI”, and “NC” respectively.

Equation (1) shows the loss function that describes how the GAN learns, where G represents the generator model and D represents the discriminator model^16^. As the goal of the GAN is to maximize the number of correctly classified sample images, the generator and discriminator models will both continuously learn through the loss function until equilibrium is reached.

1 $$\begin{array}{*{20}c} {min} & {max} \\ G & D \\ \end{array} V\left( {D, \, G} \right) = {\mathbb{E}}x\sim p_{{{\text{data}}}} (x) \, \log \, D(x) + {\mathbb{E}}{z}\sim p_{{z}} ({z}) \, \log \, (1 - \, D(G({z})))$$

#### Classification with pretrained CNN

The diagnosis and classification of MRI images into multiple stages of AD will be performed using a convolutional neural network (CNN) classifier. A CNN is a popular deep learning network used for image analysis because it can learn features directly from raw data without explicit feature engineering. Thus, it can capture rich feature representations of the data and thus achieve high accuracy in pattern recognition, identification, and classification tasks^21^. In a CNN, first, input images go through convolutional layers for feature extraction, where each layer contains a filter that activates certain features from the images. Then, in the pooling stage, the activated features are simplified, and the network parameters are reduced through weight sharing. Finally, in the classification layer, the fully connected layer outputs the probabilities of the classes after the input images are classified. In this study, the MRI images were classified into three (3) classes: AD, MCI, and NC.

Subsequently, the efficiency and widespread usage of CNNs in image analysis tasks have led to the development of enhanced CNN-based pretrained networks, such as AlexNet, VGG, ResNet, DenseNet, and EfficientNet. These networks were developed using transfer learning, a technique that can transfer the knowledge learned from one task to another^22^. These models were previously trained using ImageNet, a large dataset consisting of 1000 different classes of images. Then, using the knowledge and model parameters learned from the previous task, these pretrained CNN models can be simply used for another task with just some fine-tuning. Using pretrained networks can save time and computational resources when designing a brand-new deep learning model for a task while achieving exceptional results due to rich knowledge^23^. Numerous studies have utilized pretrained CNN models for AD classification tasks and achieved high performance^24–26^.

**Figure 4:**
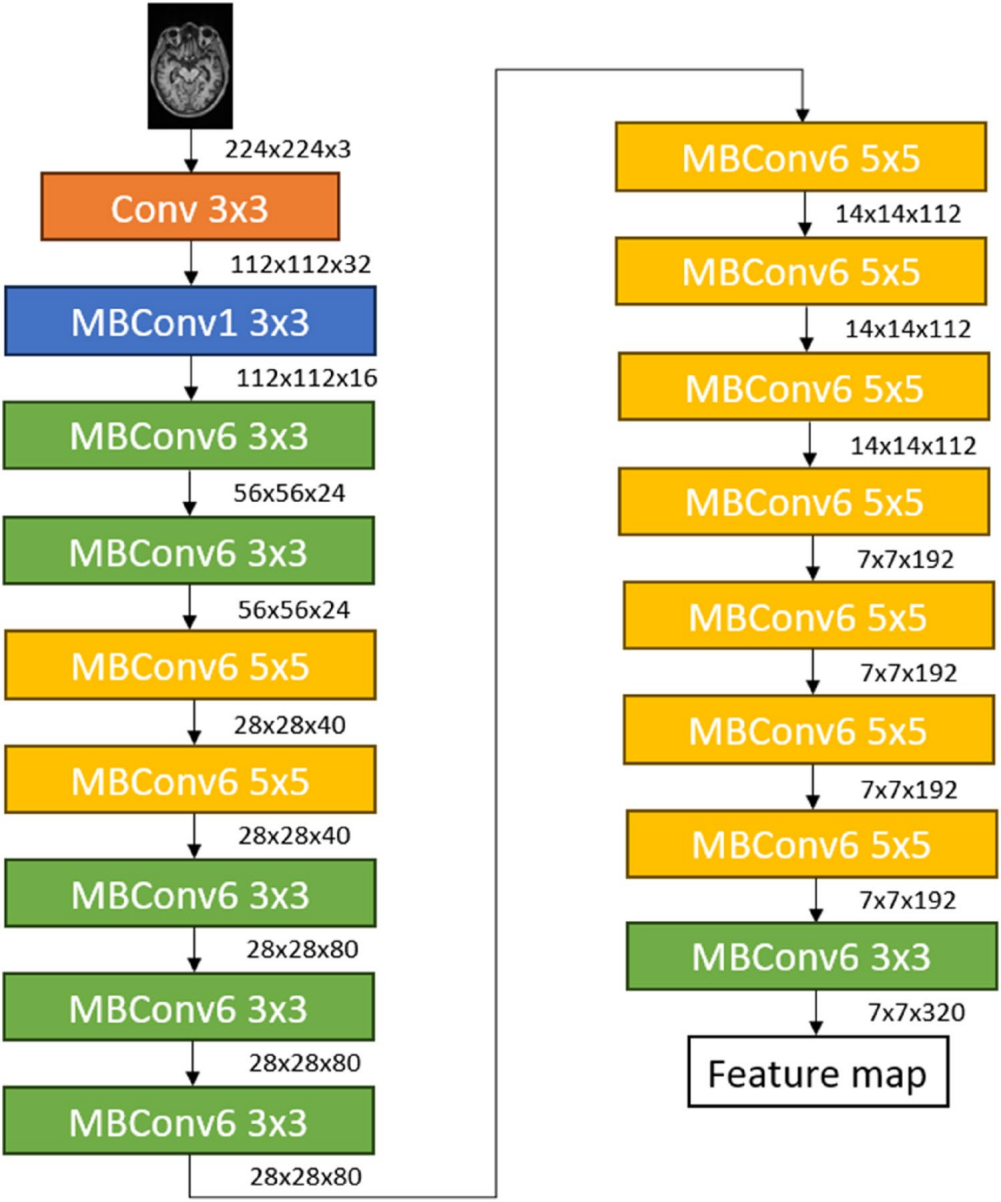
EfficientNet model architecture^28^.

In this study, the pretrained EfficientNet model was chosen to classify MRI images into different stages of AD. The EfficientNet model balances accuracy and computational resources^27^. It can achieve higher accuracy with fewer parameters and calculations than other networks due to its ability to efficiently adjust the network's size and resolution. Figure 4 shows the network architecture of the EfficientNet model. It has an efficient network design that is made up of 2D depthwise convolutional units^28^. The network mainly consists of mobile inverted bottleneck convolutions (MBConv), and this structure is useful for optimizing computational resources in EfficientNet.

### Experiment setup

As the proposed methodology shown in Fig. 2 has two significant steps, this study will perform two experiments to achieve the proposed objective: use a GAN to achieve the same accuracy with less data. Experiment 1 synthesized MRI images using a GAN, while Experiment 2 involved multistage classification of MR images using the EfficientNet model. The GAN and EfficientNet models were developed in these two experiments using the Python-based deep learning framework PyTorch and a graphics processing unit (GPU). Commonly used for research development and implementation, PyTorch is an open-source machine-learning library that emphasizes automated differentiation, tensor computation, and GPU acceleration^29^. The experimental setups for both experiments are discussed in the following subsections.

#### Experiment 1

An experiment to generate synthetic MR images using a GAN is performed to test and validate the objective proposed in this study: to achieve the same accuracy with less data. By training the GAN using real MR images obtained from the OASIS database, synthetic or fake MR images can be produced, and these data will be applied in Experiment 2. Table 2 shows the hyperparameters assigned for training the GAN in this experiment.

**Table 2:** Summary of the hyperparameters used for GAN training.

Hyperparameters	GAN training
MRI image size	64 × 64 × 3
Number of training data	AD: 1000
	MCI: 1000
	NC: 1000
Size of latent vector, z	100
Epoch	100
Batch size	64
Learning rate	0.0002
Optimizer	Adam optimizer
Loss function	Binary cross entropy

After being resized to dimensions of 64 pixels by 64 pixels for the RGB images, the 1000 MR images per class collected from the OASIS database were input into the GAN model for training. The size of the latent vector, the number of epochs, the batch size, the learning rate, the optimizer, and the loss functions used to train the model are subsequently determined based on a paper by Radford et al.^30^, where these values have been identified as the ideal hyperparameters that can enable the network to output the best results. The performance of the GAN model is evaluated by observing the generator and discriminator losses, as well as the similarity between the generated MR images and the real images collected.

#### Experiment 2

In this experiment, a test of whether the classification accuracy can be maintained even with a lesser amount of real data is carried out. As explained in Table 1, the dataset collected for this study included 1000 MRI images per class. For this experiment, the dataset was divided at a ratio of 8:2 for classification model training and validation. For each class, 800 MRI images were used for model training, while the remaining 200 MRI images were used for model validation.

To verify the hypothesis of this study, different ratios of real data versus synthetic data are tested on the classification model. Different amounts of real MR images were used for each ratio, as shown in Table 3. From the previous experiment, the synthetic MR images generated by the GAN are applied in this experiment after being normalized and resized. In the first ratio (1:0), which acts as a control set, a maximum amount of real data (800) is included since the usage of the GAN is not incorporated in this set. As the amount of real data used gradually decreases, other ratios are gradually applied to the synthetic images to maintain a balanced dataset, as shown in Table 3. Different distributions of real and synthetic MR images are included according to the different ratios, and each dataset is used to train the EfficientNet model for classification.

**Table 3:** Dataset distribution.

Datasets	Real vs generated images ratio	Number of original images	Number of generated images	Total number of images
Without GAN	1:0	AD: 800	AD: 0	800
		MCI: 800	MCI: 0	
		NC: 800	NC: 0	
With GAN	9:1	AD: 720	AD: 80	800
		MCI: 720	MCI: 80	
		NC: 720	NC: 80	
	8:2	AD: 640	AD: 160	800
		MCI: 640	MCI: 160	
		NC: 640	NC: 160	
	7:3	AD: 560	AD: 240	800
		MCI: 560	MCI: 240	
		NC: 560	NC: 240	
	6:4	AD: 480	AD: 320	800
		MCI: 480	MCI: 320	
		NC: 480	NC: 320	
	5:5	AD: 400	AD: 400	800
		MCI: 400	MCI: 400	
		NC: 400	NC: 400	
	4:6	AD: 320	AD: 480	800
		MCI: 320	MCI: 480	
		NC: 320	NC: 480	
	3:7	AD: 240	AD: 560	800
		MCI: 240	MCI: 560	
		NC: 240	NC: 560	
	2:8	AD: 160	AD: 640	800
		MCI: 160	MCI: 640	
		NC: 160	NC: 640	

Table 4 shows the hyperparameters applied for the training and validation tasks of the EfficientNet model during the classification of the MRI datasets into three (3) stages of Alzheimer's disease. Using the various MRI dataset ratios, as shown in Table 3, the EfficientNet model is trained, and the results for each dataset are observed to determine whether the proposed objective can be achieved. As shown in Table 4, upon inputting the MR images into the EfficientNet model, the images need to be resized to 224 by 224  pixels dimensions to match the network architecture, as shown in Fig. 4. Then, the model is trained based on the hyperparameters, as shown in Table 4; these values are inspired by Tan et al.^28^. The performance of the pretrained EfficientNet model was evaluated based on the classification accuracy for different dataset ratios of MRI images.

**Table 4:** Summary of hyperparameters used for EfficientNet training and validation.

Hyperparameters	Training	Validation
MRI image size	224 × 224 × 3	224 × 224 × 3
Number of image data	800	200
Epoch	100	100
Batch size	32	32
Learning rate	0.0001	0.0001
Momentum	0.9	0.9
Optimizer	Adam optimizer	Adam optimizer
Loss function	Categorical cross-entropy	Categorical cross-entropy

## Results

### Experiment 1

**Figure 5:**
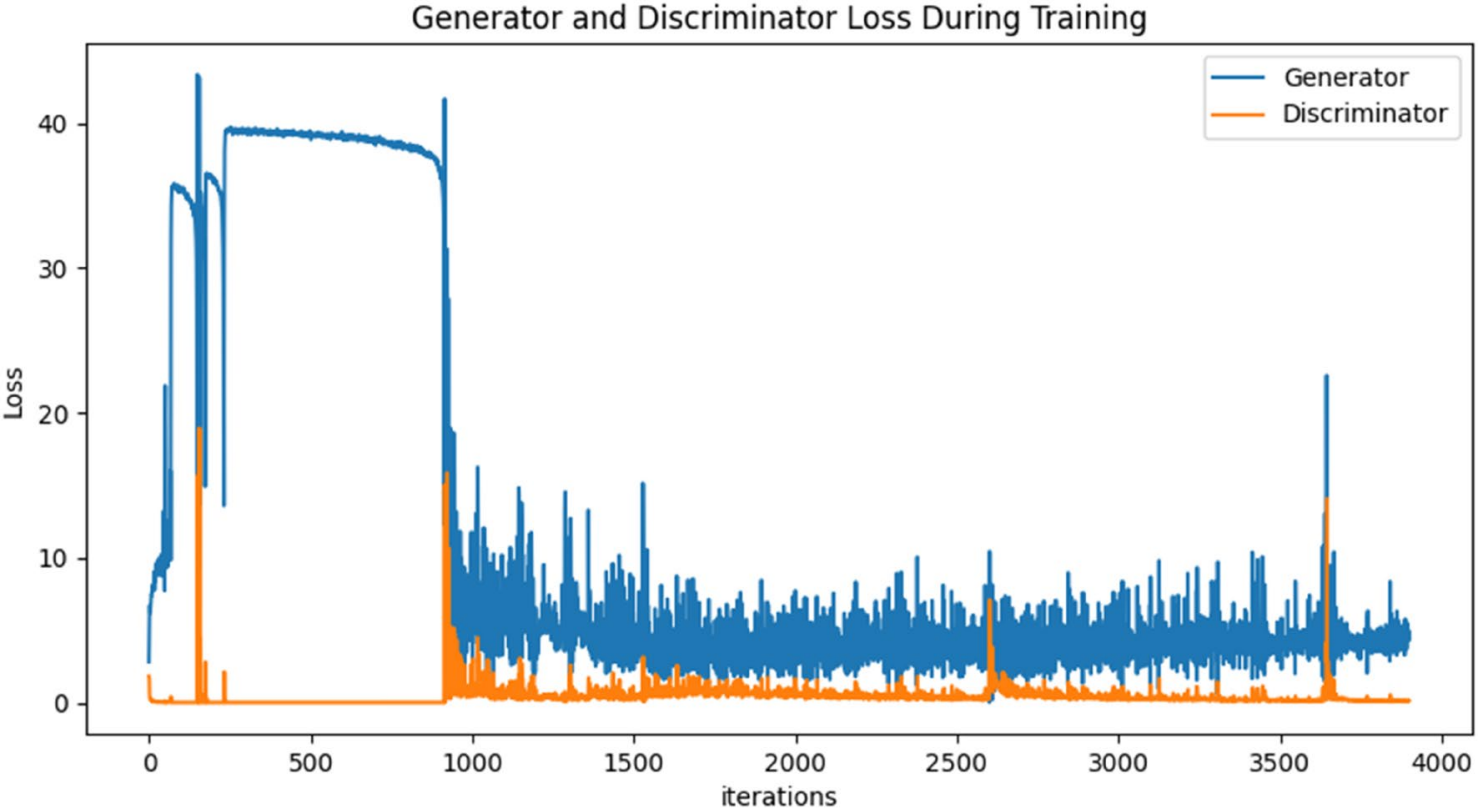
Performance of the GAN model.

In this experiment, a GAN is applied to generate synthetic MR images based on real data obtained from OASIS. The objective of this study was tested and validated, in which the image data produced in this experiment were used to facilitate Experiment 2. Figure 5 shows the performance of the GAN in terms of its generator and discriminator losses during training. The generator loss illustrates how successfully the generator generates realistic sample images and is determined based on the discriminator's reaction and feedback. On the other hand, the discriminator loss indicates the ability of the discriminator to distinguish between real and fake images. In a GAN, the generator and discriminator models are always in a competitive environment where they are concurrently trained based on game theory. One tries to generate realistic samples to trick the other, while the other tries to discriminate the reals and fakes as accurately as possible. Thus, the training of a GAN will be completed when both the generator and discriminator reach an equilibrium point. In the loss graph, as shown in Fig. 5, the equilibrium is represented by the convergence of both network models to nearly constant values as the number of iterations gradually increases. According to the training procedure performed in this study, an accuracy of 97.36% is achieved when training the GAN to generate synthetic MR images.

In addition, some comparisons between real and synthetic MRI images for each class of Alzheimer's disease have been carried out. A comparison table is shown in Table 5, where one example of real and generated image from AD, MCI, and NC class is included in the table. From Table 5, the structures and details of the MRI images can be clearly viewed and compared between real and generated data. The figures show that the GAN performed exceptionally well in the generation of realistic MR images. The generated synthetic images have similar edges and detailed structures that look as realistic as the real images. Hence, the GAN successfully produced the image data needed to facilitate Experiment 2, a crucial step in achieving the proposed objective in this research.

**Table 5:** Comparison between real and GAN-generated MR images for each class.

Class	AD	MCI	NC
Real images			
Generated images			

#### Experiment 2

Following the success of Experiment 1 in facilitating this experiment, a pretrained EfficientNet model was used to classify MRI images into multiple classes of ADs. As shown in Table 6, nine (9) balanced datasets with different ratios of real versus synthetic MR images were used in this experiment to test the model's performance in multistage classification. This experiment aimed to test whether the classification accuracy could be maintained by decreasing the number of real MR images. The function of incorporating GAN-generated images in this experiment is to maintain a balanced dataset for each ratio and avoid bias in the results.

**Table 6:** Classification results of the EfficientNet model.

Datasets	Real vs generated images ratio	Training accuracy (%)	Training loss (%)	Validation accuracy (%)	Validation loss (%)
Without GAN	1:0	99.083	0.023	88.667	0.948
With GAN	9:1	99.542	0.015	87.167	0.994
	8:2	99.625	0.012	82.500	1.262
	7:3	99.667	0.014	80.167	1.319
	6:4	99.375	0.020	75.000	1.378
	5:5	99.458	0.015	73.167	2.063
	4:6	99.958	0.013	68.000	2.128
	3:7	99.458	0.028	61.167	2.201
	2:8	99.917	0.014	58.500	2.468

In the first dataset, with a ratio of 1:0, all the MR images are real data obtained from the OASIS database, without using a GAN. This dataset will serve as the benchmark for this experiment to observe the performance of the EfficientNet model in the following cases where less real data is used. With respect to the control set, the training accuracy of EfficientNet for multistage classification reaches 99.083%, while for validation, an 88.667% accuracy is achieved.

Then, the GAN-generated MR images are gradually included in the following datasets with different ratios because a lesser amount of real data is used in these datasets. For example, in the 8:2 dataset, only 80% of the total real images are used, while another 20% of synthetic images are included to maintain a balanced dataset. In other words, when the benchmark dataset with a ratio of 1:0 includes 800 real MR images, the 8:2 dataset includes only 640 real images. By observing its classification results in this case, 99.625% accuracy was obtained from training, while validation resulted in 82.500%. Table 6 shows that the validation accuracy achieved by the first four ratios is as high as 80%. Thus, as compared with the control set, the datasets with ratios of 9:1, 8:2, and 7:3 achieved remarkably high accuracies even with less real data used.

In the other remaining datasets, the amount of real MRI data used gradually decreases as more synthetic images are added. The classification accuracy results for both training and validation are tabulated in Table 6. As observed, the validation accuracy for the datasets with ratios of 6:4 and below slightly decreases compared to that of the control set. For visual representation purposes, the results in terms of accuracy and loss graphs are also illustrated for a few of the dataset ratios, as shown in Table 7, 8, 9, 10, 11. Table 7 shows the accuracy and loss graphs for the control set, while Table 8, 9, 10, 11 show the results for datasets with different amounts of real and synthetic MR images used.

**Table 7:**
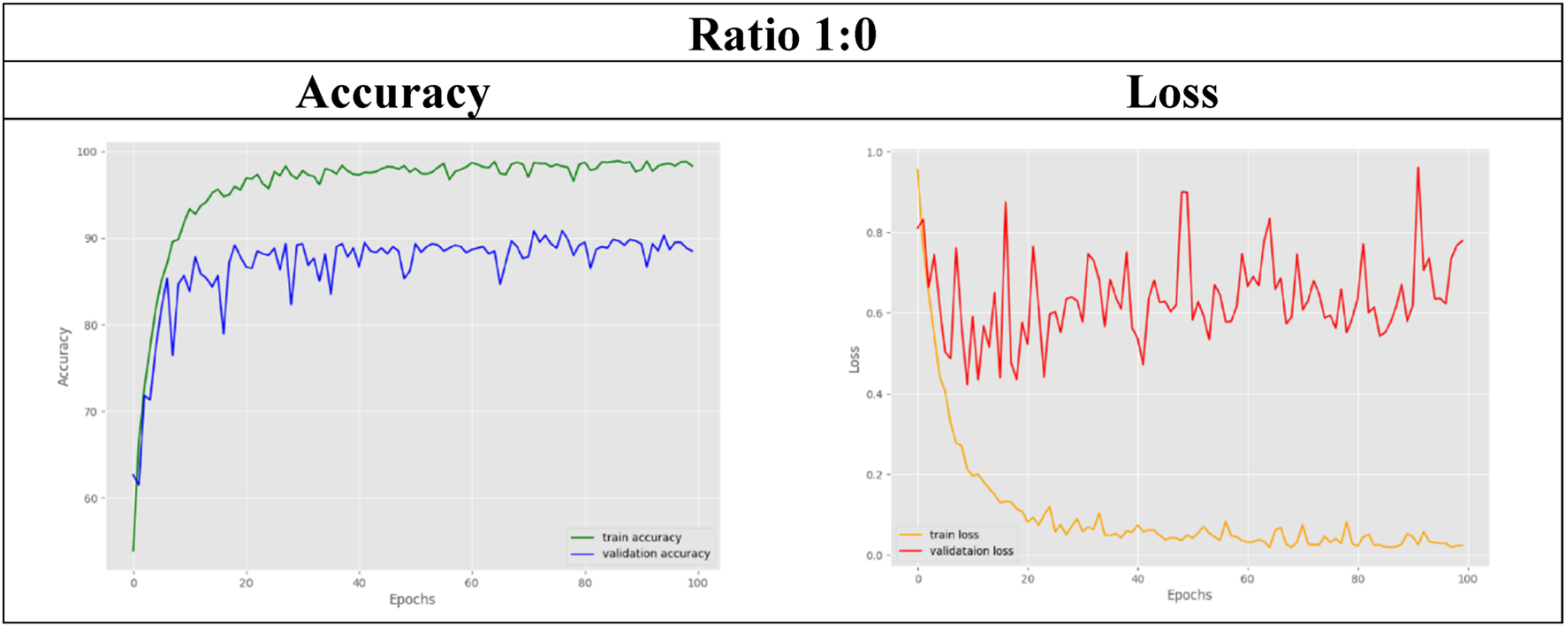
Accuracy and loss graph for a dataset ratio of 1:0

**Table 8:**
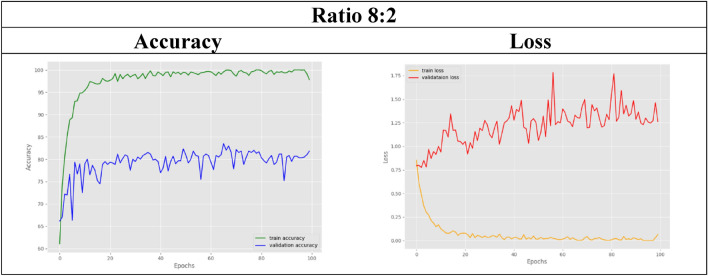
Accuracy and loss graph for a dataset ratio of 8:2

**Table 9:**
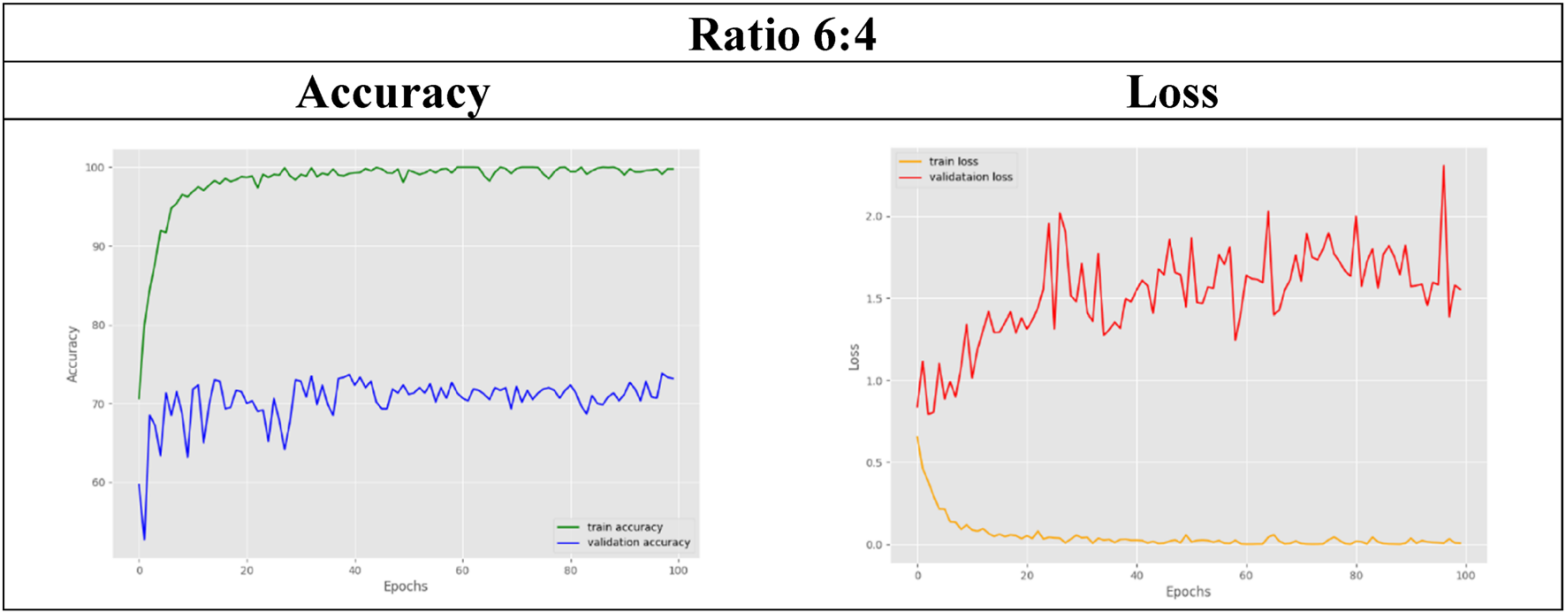
Accuracy and loss graph for a dataset ratio of 6:4

**Table 10:**
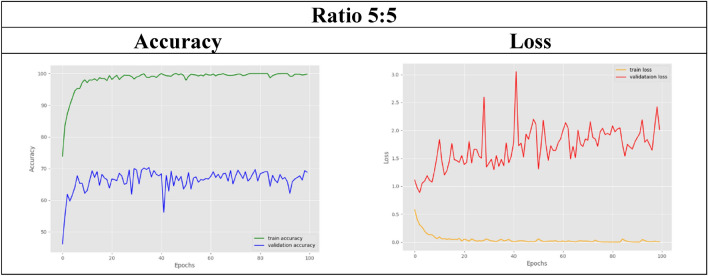
Accuracy and loss graph for a dataset ratio of 5:5

**Table 11:**
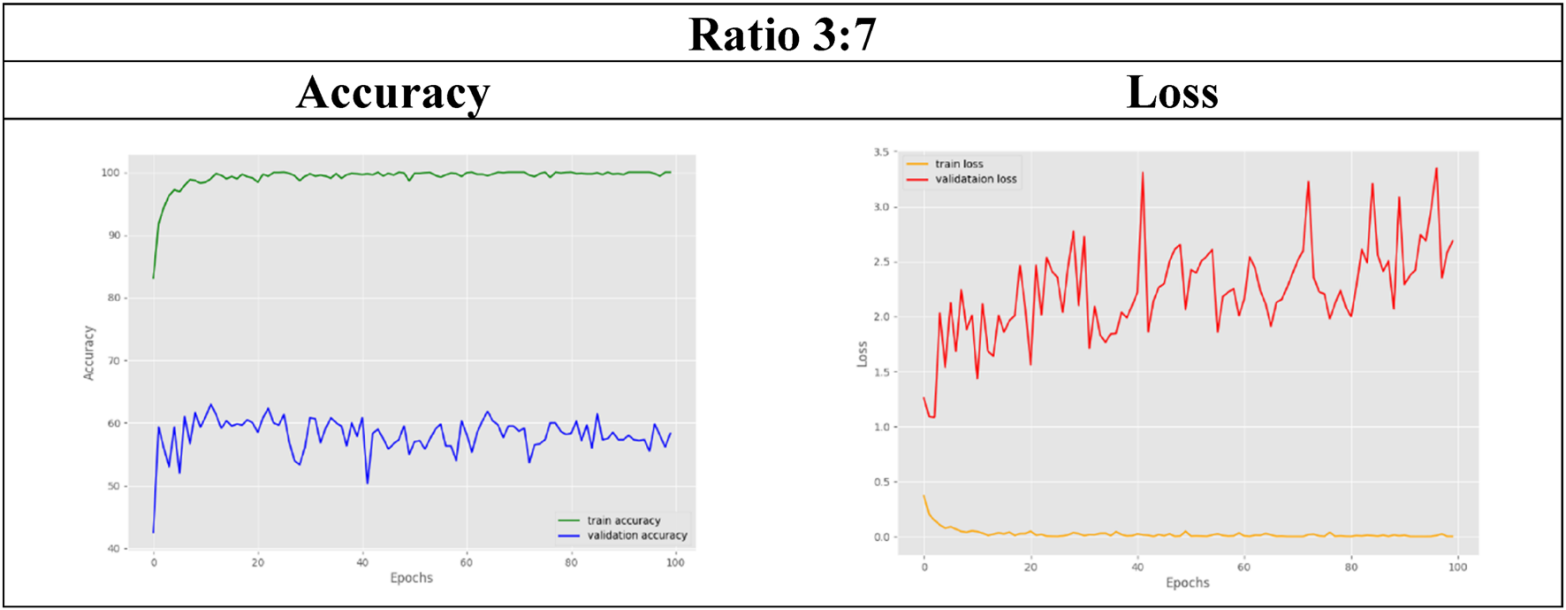
Accuracy and loss graph for a dataset ratio of 3:7

## Discussion

Based on the results of Experiment 1, the GAN model successfully generated synthetic MR images based on the real data input. By achieving an accuracy of 97.36%, the generated images are quite similar to the real data, as shown in the comparison figures. The GAN model efficiently learns the essential aspects and features in MR images to generate realistic images. However, as observed from the generator and discriminator loss graph, this experiment still has several limitations. During the beginning of the training process, the loss values are quite unstable and fluctuate at high values. After training for approximately 1000 iterations, the stability and accuracy increase as the loss values are maintained at low values. For an ideal GAN, the generator and discriminator losses are supposed to converge to a value where perfect equilibrium is reached. However, due to the instability and difficulty in training a generative model, such a perfect equilibrium is usually impossible to achieve. In contrast, convergence to a steady point is normally achievable and is sufficient for good-performance GANs. Thus, as observed from the graph in Fig. 5, both losses converge to a steady value but do not reach the perfect equilibrium point. However, despite these limitations, the generated synthetic images used in this study are quite accurate and sufficient for facilitating classification.

On the other hand, experiment 2 successfully utilized the pretrained EfficientNet model to classify MRI images into multiple stages of Alzheimer's disease. Nine datasets were used to test the model's classification accuracy when trained with different amounts of MR images. Starting from 100%, 90%, 80%, and so on, different percentages of the MRI images collected from the OASIS database are used in the experimental datasets. Dataset 1, which uses 100% real MR images, acts as the control set, while starting from dataset 2, only 90% of the real images are used, and this percentage decreases gradually for the other datasets. To maintain a balanced dataset in all the datasets, insufficient or vacant datasets with reduced real images were loaded with synthetic MR images generated by the GAN in experiment 1 instead. After the EfficientNet model is trained for multistage classification, the training accuracy achieved for all the datasets with or without the use of a GAN is maintained at approximately 99%. The classification model was subsequently validated with different dataset ratios, and comparable results were obtained, as shown in Table 6. Compared with the control set, the validation accuracies obtained by the datasets with ratios of 9:1, 8:2, and 7:3 were comparable at approximately 80% and above. Thus, this comparable accuracy can be used to validate the hypothesis that the EfficientNet model can produce the same classification accuracy even with fewer data used. This study has several limitations. As shown in Table 6, the datasets with a smaller ratio of real images than generated images experienced a slight decrease in their validation accuracies compared to those of the control set. This might be due to too many generated images being included in the dataset, which included only a small number of real images. Therefore, the accuracy slightly decreases because the test set used for validation comprises only real MR images. Hence, since the model was trained on a large number of generated images, it has limited ability to identify real images. Nevertheless, the classification accuracies achieved by the model were still acceptable, as they were still maintained at approximately 60% and above, even for the dataset with the slightest real data used. Hence, from the results obtained, it can be inferred that the 9:1, 8:2, and 7:3 ratios of real versus synthetic images are the best and ideal situations where fewer real images can be obtained from the database while at the same time achieving the same accuracy as the benchmark dataset.

## Conclusion

In summary, the objective proposed in this study was successfully achieved, albeit with several limitations. The aim was to utilize a GAN to attain comparable accuracy with reduced data in AD classification. The results from both experiments demonstrate that the exceptional performance of the EfficientNet model in AD classification can be maintained even when utilizing a smaller MRI dataset obtained from the OASIS database for training. Therefore, by validating this hypothesis through these experiments, the challenge of insufficient data in AD classification can be effectively addressed by incorporating a GAN. This study contributes valuable insights to the research field and can potentially enhance AD diagnosis for the well-being of Alzheimer's disease patients. Future research directions may involve improving the stability of GANs, enhancing the generalization ability of the EfficientNet model, and incorporating additional enhancement modules to further enhance classification performance.

## Data Availability

The MRI dataset was collected from the Open Access Series of Imaging Studies (OASIS) (https://www.oasis-brains.org/).
